# Eyes wide open: an essay on developing an engaged awareness in global medicine and public health

**DOI:** 10.1186/s12914-014-0029-4

**Published:** 2014-10-28

**Authors:** William B Ventres, Meredith P Fort

**Affiliations:** 1 Institute for Studies in History, Anthropology, and Archeology, University of El Salvador, Urbanización Buenos Aires III, Block H, Calle Los Maquilishuat N° 3-A, San Salvador, El Salvador; 2 Department of Family Medicine, Oregon Health and Science University, Portland, OR, USA; 3 Institute of Nutrition of Central America and Panama, Guatemala City, Guatemala

**Keywords:** Epidemiologic factors, Health knowledge, Attitudes, Practice, Internationality, Public health, Role, Professional, Social medicine

## Abstract

**Background:**

There is a growing understanding of the role social determinants such as poverty, gender discrimination, racial prejudice, and economic inequality play on health and illness. While these determinants and effects may be challenging to identify in parts of high-income countries, they are patently obvious in many other areas of the world. How we react to these determinants and effects depends on what historical, cultural, ideological, and psychological characteristics we bring to our encounters with inequity, as well as how our feelings and thoughts inform our values and actions.

**Discussion:**

To address these issues, we share a series of questions we have asked ourselves¿United States¿ citizens with experience living and working in Central America¿in relation to our encounters with inequity. We offer a conceptual framework for contemplating responses in hopes of promoting among educators and practitioners in medicine and public health an engaged awareness of how our every day work either perpetuates or breaks down barriers of social difference. We review key moments in our own experiences as global health practitioners to provide context for these questions.

**Summary:**

Introspective reflection can help professionals in global medicine and public health recognize the dynamic roles that they play in the world. Such reflection can bring us closer to appreciating the forces that have worked both for and in opposition to global health, human rights, and well-being. It can help us recognize how place, time, environment, and context form the social determination of health. It is from this holistic perspective of social relations that we can work to effect fair, equitable, and protective environments as they relate to global medicine and public health.

## Background

The influence of social determinants on global health has been well documented [1],[2]. Poverty, gender discrimination, racial prejudice, and economic inequality are four of many determinants that put people at risk for poor health-related outcomes [3]. Throughout the world, the ¿have nots¿ struggle to acquire and benefit from the same things that keep the ¿haves¿ healthier, including access to potable water, opportunities for dignified work, and the right to decent education and reasonable medical care [1],[4]. For some, this situation reflects structural violence: a normalized albeit almost invisible state of social injustice inherent in the organization and governance of both public and private institutions [5]. For others, it is business as usual: there have been winners and losers for centuries all around the world [6],[7].

What is new, at least in the collective consciousness of medicine and public health, is an emergent understanding of both the roles these determinants play and the dimensions of their effects [8],[9]. This understanding is in part due to a mounting sense of shared vulnerability [10]; infectious diseases such as HIV/AIDS, SARS, Ebola, and dengue fever cross international borders and threaten people in low- and high-income countries alike [11]. It is due to a growing attention given to the cross-national migration of capital, people, and products (as well as their means of production) [12]¿[14]. It is due to a conviction that the world is an ever-smaller place because of increasing population density, rising rates of international migration, and the rapidly accelerating pace of information transfer [4].

Social determinants of health and their effects are patently evident in places such as El Salvador and Guatemala, where we¿the authors, both United States¿ citizens¿live and work. In these two countries, as in many other low- and middle-income countries, vast wealth and miserable poverty literally collide: high-class condominiums and boutique shops face newly renovated roads while corrugated tin shacks fill in the neglected areas that line their backyards. One cannot help but see the stark consequences of these troubling realities.

However, what seems not so evident is the process by which we as medical and public health professionals from high-income countries incorporate such observations into our consciousnesses [15].

 How do we comprehend differences between what we are accustomed to in our countries of origin and what we see in other places around the world?

 What do we make of the immense divisions that exist between and within societies?

 How is it that we open ourselves to hear the stories born of these divisions, and how do we integrate them into our daily lives and personal histories?

 Why is it that some can see, reflect on, and address the causes and consequences of social determinants, while others cannot or choose not to do so?

How each of us perceives, explores, and acts upon social determinants and health disparities depends on many factors, including historical, cultural, ideological, and psychological characteristics, along with other influences such as ethnic heritages, professional socializations, and families of origin [16]¿[18]. Our cultural and social baggage shapes how we view and understand the problems we see; it also guides whether and how we choose to address them. Regardless of whether we are new to the field of global health or old hands in its study, teaching, and practice, our willingness to witness and attend to issues of social determinants and health disparities depends on our ability to ask deep and thought-provoking questions.

In this article, we share questions we have asked ourselves in the face of obvious inequity, and review, in two short vignettes, key moments in the development of our self-awarenesses. We share these questions and vignettes to model our own processes of reflection. We do so hoping that global health professionals might become more conscious of the burdensome realities of social determinants and their consequences. We do so, as well, hoping that global health professionals, especially those from high-income countries, might begin to examine thoughtfully how their every day work either perpetuates or breaks down barriers of social difference. We believe such examination can enhance capacities to work toward ameliorating inequities in ways that are supportive, sustainable, and satisfying.

## Discussion

### Asking questions in global health education and practice

Common questions can be asked of all those engaging in the work of global medicine and public health.

 What are our interests in and motivations for exploring beyond the relatively comfortable boundaries of training and practice at home?

 What are our preconceptions built from personal and professional history, previous socialization, and sense of self in relationship to others?

 How do we recognize our preconceptions when confronting that which is new and different?

 How do we manage these preconceptions when working with peers in low-resource settings?

 Do our cultural upbringings enable or inhibit us from seeing underlying social factors that contribute to poor health?

 Both at work and in our home communities, how can we productively contribute to minimize community-level health disparities?

Much, too, depends on how we experience our responses in the face of social determinants and social inequities.

 Do we sense fear or anxiety in ourselves?

 Are we profoundly shocked, indignantly outraged, merely uncomfortable, or at ease with it all?

 Is anger mixed in, or pity, or rejection, or any one of numerous other possible feelings?

 Are we even aware of our emotional responses? How do we balance the clinical detachment of our professional socializations and the profound need for empathy in times of suffering?

 Alternatively, are we simply overwhelmed by encounters with social problems and thus not able to absorb the cognitive and emotional confusion that often accompanies witnessing them?

 If we see work in global health as a reciprocal undertaking, one in which we both give and receive, teach and learn, nurture and grow¿and we acknowledge that not all share our view in this regard¿can we sense our interconnectedness with the workings of the world around us?

### Cultivating a personal consciousness

Our responses in turn depend on how feelings and thoughts inform our values. Each of us enters into global health work holding ingrained values based on previous experiences. It would be unusual were we not to challenge our values in the face of new wisdom gained from the voices, words, and actions we hear, read, and see in our ventures domestically and abroad. It would be surprising to see people living in situations vastly different than those which we are accustomed to and not expect some change in these values. It would be an opportunity missed were such experiences not to enhance our capacities to act with authenticity and integrity.

We believe that understanding and addressing the social determinants of health and health disparities starts by contemplating questions such as those above. We encourage practitioners, educators, and learners in public health and medicine to ask these questions of themselves and reflect on them before, during, and after global work experiences. Only by opening ourselves to such self-dialogue can we develop abilities to see others from positions of equity and solidarity, rather than from dominance and exploitation. Only by probing deeply into our own personal consciousnesses can we know others as real people living in real situations, rather than as intellectualized variables in uncontrolled environments. Only by examining individual presences relative to the much larger political, economic, ideological, and social forces (many of which have created environments that frame the very problems we aim to eliminate) can we effectively address pressing issues of disease management [19]. Even if we enter into our global health endeavors with good intentions¿and we are inclined to believe that few in the healing professions do not¿by avoiding such introspection we limit our capacity to see and understand, with eyes and minds wide open, the reality and meaning of life concerns as they are seen from perspectives of those who are foreign to us.

### Developing an introspective gaze in global health

What new understandings might such an introspective gaze offer us? First, we believe such a gaze helps us assume postures of inquiry rather than expertise. Regardless of what knowledge we might already possess, we also have much to learn by asking, questioning, and wondering. Such a gaze allows us to begin by scratching our heads, with interest in discovery and intention to comprehend, before contemplating physical diagnosis or program implementation [20]. Demonstrating a humble curiosity¿defining humble as respectfulness not subservience, and curiosity as genuine concern not academic nosiness¿broadens our outlooks beyond comfort zones based on professional status or sphere of knowledge [21],[22]. It improves our skills in crossing borders of culture and class. It enhances our abilities to learn from the lived experiences of colleagues, friends, and scholars (Additional file 1: Personal Vignette 1).

Second, an introspective gaze helps us see our place as participants in systems that extend across dimensions of time, ethos, politics, and geography [23]. As global health professionals, our work inevitably touches on contextual issues such as culture, history, power, and ideology [24]. This does not mean we are complicit in the wrongs of the world or support the collateral damage that results in the wake of many policies. It does mean we are all actors on the set, whether with speaking roles or simply standing by as extras. The recognition of our presence in these complex systems can help us judge our capacities for innovation, collaboration, and action accurately and reliably. Specifically, the work we choose and the way we choose to do it helps us either break or recreate historically produced patterns of inequity.

Third, this gaze helps us perceive how we bring to our interactions something uniquely ours, and how each of us reciprocally shares our unique nature with others. Through this process, our relationships with others become less ¿us and them¿ and more ¿we¿ [25]. We see how interdependent our lives are. We share common wounds and resiliencies, even as their depths and extents may vary. We share similar hopes and dreams, even as their expressions may be remarkably different. We share capacities for growth and intimacy, as well as the reality that both joy and tragedy will assuredly touch all our lives (Additional file 1: Personal Vignette 2).

Fourth, it helps us keep aware of where we have been, literally and figuratively, in our personal and professional journeys. Working as professionals in global health, we become increasingly cognizant that concepts like core and periphery, dominant and subservient discourses, and oppression and marginalization are not just matters that exist elsewhere [24],[26]. They exist everywhere [27]¿[29]. With this awareness comes the conceptual realization that local and global are not so far apart: there is work to be done in both locales [30],[31].

Fifth, an introspective gaze helps us appreciate our potential to act as change agents. It helps us hear and cultivate our own voices as advocates at the same time we hear and cultivate the voices of others. At a time when many are calling for reform in the delivery of medical care around the world [32],[33], it helps us develop resiliency in the face of political resistance to innovation [34]¿[37]. It helps us balance empathy, understanding, and action in our work as global health professionals.

Finally, such a gaze helps us recognize the dynamic role that each of us plays in the world. This role is not simply about examining and attending to social determinants or their end results; it is about understanding forces that work both for and against health and well-being, molding conceptions of place, time, nature, and people¿including ourselves¿into an integral notion of the social *determination* of health. Social determination of health is a framework, born of Latin American scholarship, that has shaped how we approach our work in global health [38],[39]. In particular, it has informed our belief that the establishment of fair, equitable, and protective health environments comes through a collective construction of profound social awareness, one in which each of us recognizes that our everyday actions can either promote or inhibit social equity.

## Summary

As we work to ameliorate inequities and improve health outcomes, we as educators and practitioners in global medicine and public health confront many challenges. Among them are unconscious assumptions that can blind us to the realities of others¿ suffering and limit the breadth of our personal visions. Among them are learned convictions that may hinder our abilities to perceive those things that bind us together rather than rip us apart. Among them, as well, is the ordeal of staying motivated so as not to become indifferent in the face of ongoing daily frustrations.

Wherever we may be, amidst these challenges, lies a place between self, others, and the larger geopolitical world where differences are recognized and commonalities explored. This is the terrain¿between individual and community, between particularized ego and collective consciousness, between local and global¿upon which we can learn to grow in a wisdom co-created from shared experience. This is the fertile ground on which we can nurture an engaged awareness in global health, one that promotes personal reflection, social consciousness, and communal action in efforts to acknowledge the effects of social determinants and improve health outcomes around the world.

## Competing interests

The authors declare that they have no competing interests.

## Authors¿ contributions

WBV and MPF jointly conceived of this essay, participated in its design, and contributed to its writing. Both authors read and approved the final manuscript.

## Authors¿ information

WBV is a family physician and medical anthropologist. He is a Research Associate at the Institute for Studies in History, Anthropology, and Archeology at the University of El Salvador. Besides qualitative research in public health and bioethics, his interests lie in physician-patient communication and the global development of primary care. MPF is a public health practitioner and researcher with over fifteen years experience in Central America. She is an Affiliate Researcher at the Institute of Nutrition of Central America and Panama in Guatemala City, where she recently completed a post-doctoral fellowship on the evaluation of chronic disease interventions. She is an advocate for just development policies including the promotion of primary health care and access to essential services.

## Additional file

## Supplementary Material

Additional file 1: **Personal Vignettes.** Personal Vignette 1: Connected in El Salvador. Personal Vignette 2: Inequity at a Guatemalan intersection. Click here for file
