# Supplementary material for: Eyes wide open: an essay on developing an engaged awareness in global medicine and public health
Source: BMC Int Health Hum Rights. 2014 Oct 28;14:29. doi: 10.1186/s12914-014-0029-4 (PMC4422227; doi:10.1186/s12914-014-0029-4)
Supplement: Additional file 1: — Personal Vignettes. Personal Vignette 1: Connected in El Salvador. Personal Vignette 2: Inequity at a Guatemalan intersection. [file s12914-014-0029-4-S1.doc]

Additional file 1

**Personal Vignettes**

**Personal Vignette 1: Connected in El Salvador**

I came to El Salvador in late 2010 to teach public health. I didn’t know much about the country when I arrived. I had some knowledge of the 1980’s civil war because I trained in Tucson, Arizona, where the “Sanctuary Movement”—people dedicated to providing assistance to Salvadoran victims of violence—was active. I am embarrassed to admit it, but as a medical student and resident I was just trying to keep my head above water clinically. Since then I have been busy practicing family medicine, raising kids, and managing the demands of everyday life.

Upon arriving in El Salvador, I learned many things. I learned that this is a country of huge inequities. Once 14 families, *Las Catorce*, owned most of the land at a time when land and the cotton, coffee, or sugar grown on it were the currency of trade and power. Now, it is said, with the advent of a more “open” political environment, the number has grown to forty or so.

I learned that this is a country of faith. Born of 500 years of colonization by one country or another, during which its land and natural resources have been systematically expropriated, faith is what is left for people to embrace as their own. I learned, as well, that the current “product” for exportation is people: almost one third of Salvadorans live outside the country, most in the United States.

In the U.S., people write about informal economies, globalization, and migration. In El Salvador, these are lived. Almost everyone has a family member or friend working “there” (as the U.S. is referred to “here”), with or without legal papers. Look down any major street in San Salvador and witness how forcefully the U.S. market has penetrated the Salvadoran economy: fast food restaurants with names common to North Americans—Burger King, Wendy’s, McDonalds—line them. Approximately one-sixth of the country’s Gross National Product consists of remittances from Salvadoran émigrés, and DR-CAFTA (the Dominican Republic-Central American Free Trade Agreement) has assured that free-trade zones and sweatshop-like factories (*maquilas*) are spread around the country.

I have come to recognize that global health and social determinants encompass realities where national boundaries are blurred in terms of where people live, work, raise their children, and get their care. In academic endeavors we often make "within" and "between" comparisons, but, for many people from countries like El Salvador, these are artificial constructs.

They are also artificial constructs for people like me, whose perspectives on relationships and work are born of personal histories and socializations in the United States. In Central America, I am understood to represent a whole host of other characteristics—notably money and power—that derive from my country of origin. Whether I am aware of these and how I use them to navigate in El Salvador, these are crucial factors that determine how, where, and with whom I make my way here. This is my reality.

It is all of ours, actually, in situations both comprehensible and confusing. We share it, together.—WBV

**Personal Vignette 2: Inequity at a Guatemalan intersection**

Driving back home from my office in the center of Guatemala City, I usually come to a stop at a corner on the Calzada Roosevelt, one of the city’s busiest streets. Many days I find the same people there.

There is an old man who stands on his head on a little mat and kicks his feet, as if he were on an upside-down bicycle. Another man walks slowly through the line of traffic, one limp leg, palms extended pleadingly. A family comes from far outside the city to sell bananas, mandarins, and other fruits. Then there are the 6 year-olds selling gum, their faces painted like clowns, trying to get a few extra *centavos* to keep their families fed or pay off whomever is “looking over” them.

There is a woman who juggles balls high up in to the air. This woman and I are probably of similar age. She balances a toddler on her back as she does her street-corner show, while my youngest is comfortably strapped into a backseat car seat. I think to myself, I am so lucky to be able to drive my children safely through the notoriously rough streets of Guatemala City. Yet, I also feel a pit in my stomach. I know it is not luck so much as privilege, the privilege of having been born into a stable middle class family in a wealthy society that has provided me opportunities I could offer my own kids.

In my work at a chronic disease research center, I believe that what I am doing may help people like the ones I meet at the intersection on my way home. Not immediately, maybe, but sometime down the line. I know that men and women with fewer resources and more stress more commonly suffer from diseases like hypertension or type 2 diabetes. While I know it is reasonable medical advice, I know it would be unfair to ask anyone stationed at the stoplight to scrape together extra change to buy medications and eat a healthier diet.

A mix of circumstance and structural reality, combined, put each of us at higher or lower risk for disease as members of society. In so many ways the global health community has made tremendous progress. Most kids are vaccinated. Women are much more likely to have healthy deliveries. We do a better job of screening for priority infectious diseases. We are very good at gathering data and conducting complex analyses.

But as practitioners of global health ever more cognizant of social differences and how they influence the health of individuals and communities, we are far from having an adequate response to issues of inequity. We have a long way to go in figuring out how to convert our discourse and genuine concern into approaches that rectify problems.

Most of the time, as I have been taught to do, I am able to step back and see how my work makes sense. On a population level, benefits come over the long-term. But on some days, especially during torrential rainstorms, I can’t help but think about how offering shelter and food to a few people at one city intersection might just be the right thing to do.—MPF
